# Cooperative Action of Fulvic Acid and Bacillus paralicheniformis Ferment in Regulating Soil Microbiota and Improving Soil Fertility and Plant Resistance to Bacterial Wilt Disease

**DOI:** 10.1128/spectrum.04079-22

**Published:** 2023-03-02

**Authors:** Xiuyun Zhao, Di Zhu, Jun Tan, Rui Wang, Gaofu Qi

**Affiliations:** a College of Life Science and Technology, Huazhong Agricultural University, Wuhan, China; b Enshi Tobacco Company of Hubei Province, Enshi, China; North-West University

**Keywords:** *Bacillus paralicheniformis*, bacterial wilt disease, fulvic acid, poly-gamma-glutamic acid, soil fertility, soil microbial community

## Abstract

Excessive continuous cropping and soil degradation, such as acidification, hardening, fertility decline, and the degradation of microbial community, lead to the epidemic of soilborne diseases and cause great loss in agriculture production. Application of fulvic acid can improve the growth and yield of various crops and effectively suppress soilborne plant diseases. Bacillus paralicheniformis strain 285-3 producing poly-gamma-glutamic acid is used to remove the organic acid that can cause soil acidification and increase the fertilizer effect of fulvic acid and the effect of improving soil quality and inhibiting soilborne disease. In field experiments, the application of fulvic acid and Bacillus paralicheniformis ferment effectively reduced the incidence of bacterial wilt disease and improved soil fertility. Both fulvic acid powder and *B. paralicheniformis* ferment improved soil microbial diversity and increased the complexity and stability of the microbial network. For *B. paralicheniformis* ferment, the molecular weight of poly-gamma-glutamic acid became smaller after heating, which could better improve the soil microbial community and network structure. In fulvic acid and *B. paralicheniformis* ferment-treated soils, the synergistic interaction between microorganisms increased and the number of keystone microorganisms increased, which included antagonistic bacteria and plant growth-promoting bacteria. Changes in the microbial community and network structure were the main reason for the reduced incidence of bacterial wilt disease. Application of fulvic acid and Bacillus paralicheniformis ferment improved soil physicochemical properties and effectively controlled bacterial wilt disease by changing microbial community and network structure and enriching antagonistic and beneficial bacteria.

**IMPORTANCE** Continuous cropping tobacco has led to soil degradation and caused soilborne bacterial wilt disease. Fulvic acid as a biostimulator was applied to restore soil and control bacterial wilt disease. For improving its effect, fulvic acid was fermented with Bacillus paralicheniformis strain 285-3 producing poly-gamma-glutamic acid. Fulvic acid and *B. paralicheniformis* ferment inhibited bacterial wilt disease, improved soil quality, enriched beneficial bacteria, and increased microbial diversity and microbial network complexity. Some keystone microorganisms in fulvic acid and *B. paralicheniformis* ferment-treated soils had potential antimicrobial activity and plant growth-promoting attributes. Fulvic acid and *B. paralicheniformis* 285-3 ferment could be used to restore soil quality and microbiota and control bacterial wilt disease. This study found new biomaterial to control soilborne bacterial disease by combining fulvic acid and poly-gamma-glutamic acid application.

## INTRODUCTION

The continuous cultivation of the same crops in the same field is called continuous cropping. With the development of the scale and intensification of crops, restricted by the cultivated land area and cultivation conditions, continuous cropping has become the main planting mode of crops in most of the main crop-producing areas in China (1). Continuous cropping leads to the reduced consumption and uneven enrichment of soil nutrients, resulting in an imbalance and deterioration of soil nutrients, and then affects the absorption of soil nutrients by plants (2, 3). Additionally, after serious soil degradation, it is easy to cause soilborne diseases, such as bacterial wilt disease and root rot disease, resulting in a large area of plant death and causing great economic losses (4). Bacterial wilt disease is caused by Ralstonia solanacearum, which infects more than 250 plant species (5).

Fulvic acid is a kind of yellowish-brown powder substance and is soluble in acid and alkali, containing hydroxyl group, amino group, and other groups, widely used in agriculture, medicine, and other fields (6). Fulvic acid has high quality and is the best active and resistant component of humic acid. Fulvic acid is rich in nitrogen and potassium and has the function of solubilizing phosphorus and improving fertilizer utilization rate. Fulvic acid has been widely utilized to amend soils with low fertility. Fulvic acid can also accelerate the formation of soil aggregates and improve soil physical and chemical properties, such as permeability, airing, and pH (7). In recent years, as a new biostimulator and humic acid soil modification agent, fulvic acid is widely used in facility agriculture, such as greenhouses, as well as in cash crops such as fruit trees; facilitates the assimilation of plant nutrients; and has achieved good application results (8). Fulvic acid induces disease resistance to Botrytis cinerea through the activation of the phenylpropanoid pathway (9). Although fulvic acid has been used in more and more crops, its use for biocontrol of bacterial wilt disease is still rarely reported. We had previously conducted preliminary studies on the application effectiveness evaluation of fulvic acid in tobacco cultivation and found that fulvic acid could effectively inhibit the occurrence of tobacco soilborne diseases, such as root rot disease. This study was based on previous research to further develop the application technology of fulvic acid in crop planting, establish a solid foundation for improving soil quality, and prevent the continuous cropping obstacle and soilborne plant diseases.

Bacillus paralicheniformis strain 285-3 was isolated from soil sample collected from Shanxi province, China. *B. paralicheniformis* strain 285-3 produced poly-gamma-glutamic acid (γ-PGA) with an average molecular weight of 580 kDa. The γ-PGA is an environmentally friendly biomaterial that has a broad application for food, medicine, agriculture, environmental protection, and other fields (10). In this study, fulvic acid powder (FA) extracted from yeast molasses fermentation was added in culture medium of 285-3 to remove the organic acid that could cause soil acidification and increase the fertilizer effect of fulvic acid and the effect of improving soil quality and inhibiting soilborne bacterial wilt disease.

## RESULTS

### Antagonism of fulvic acid and *B. paralicheniformis* ferment on bacterial wilt disease.

**FIG 1:**
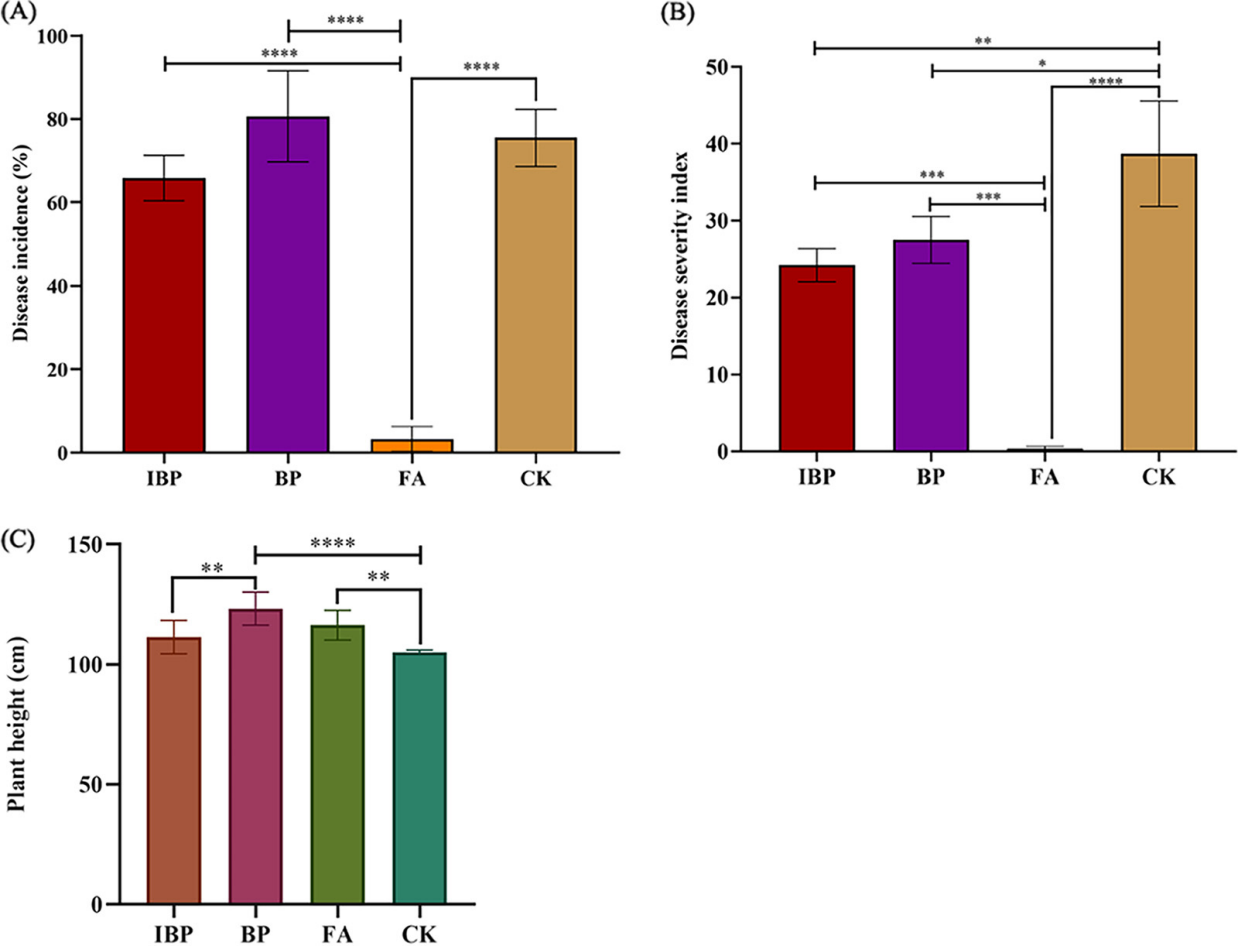
Biocontrol effect of fulvic acid and Bacillus paralicheniformis ferment on bacterial wilt disease. (A) The disease incidence of tobacco bacterial wilt in different treatments. (B) Disease severity index of bacterial wilt. (C) Plant height. *, *P* < 0.1; **, *P* < 0.01; ***, *P* < 0.001; ****, *P* < 0.0001.

Fulvic acid powder (FA) treatment significantly (*P *< 0.0001) reduced the disease incidence and disease severity index of tobacco bacterial wilt (Fig. 1). Compared with the control group (CK), the disease incidence and disease severity index in FA treatment was reduced by 95.63% and 99.05%, respectively. Disease severity index in the unheated *B. paralicheniformis* ferment (BP) and heated *B. paralicheniformis* ferment (IBP) treatments also significantly decreased compared with that of the control. Application of fulvic acid or heat-treated or untreated fermentation broth of *B. paralicheniformis* 285-3 all effectively controlled bacterial wilt disease. Fulvic acid powders exhibited the best control effect on bacterial wilt disease among all treatments. The application of fulvic acid and *B. paralicheniformis* ferment had a promoting effect on tobacco plants’ growth, among which BP and FA treatments had an obvious promoting effect on plant height.

### Improvement in soil physicochemical properties and plant growth by fulvic acid and *B. paralicheniformis* ferment.

**FIG 2:**
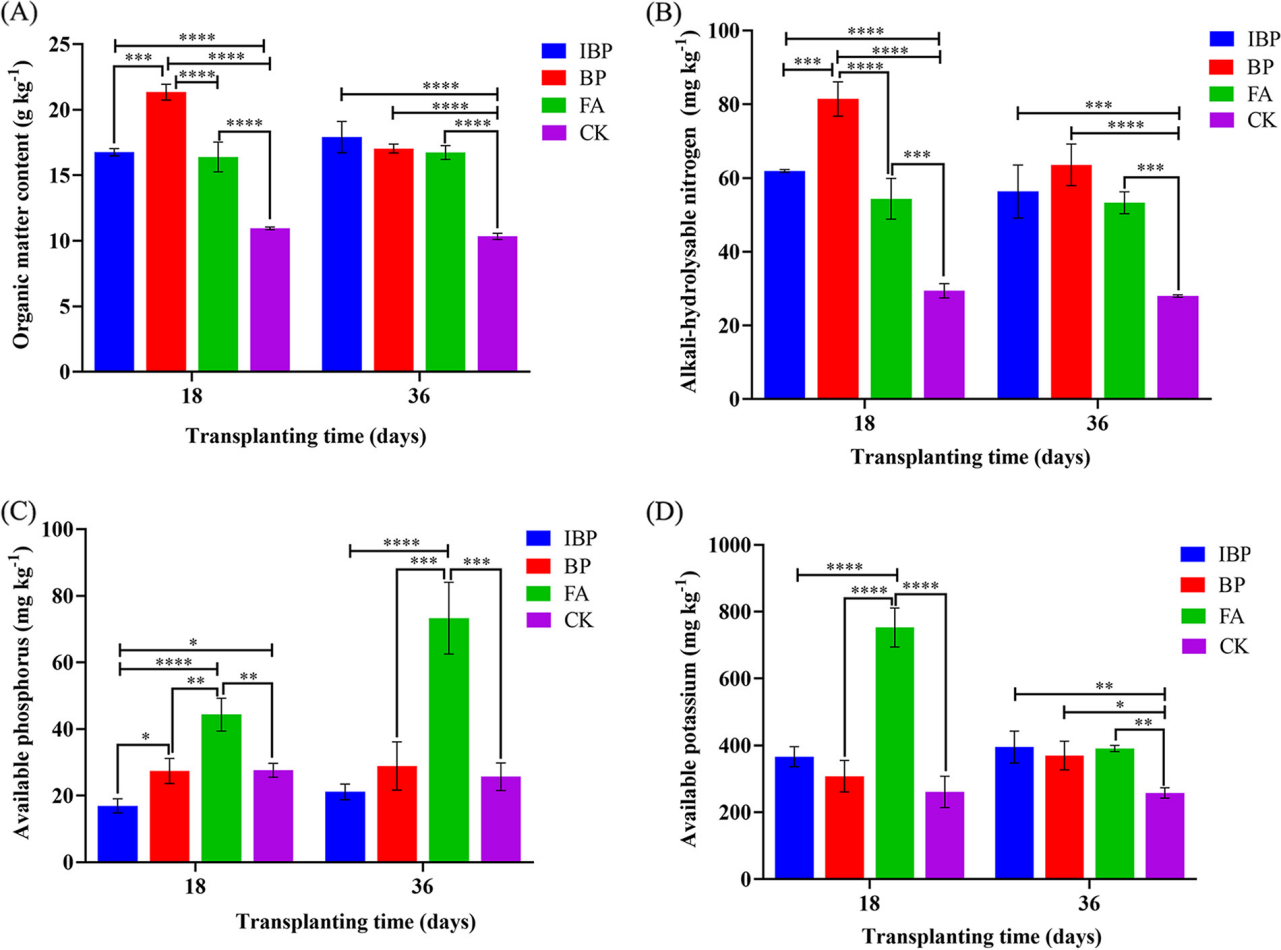
Effect of fulvic acid and Bacillus paralicheniformis ferment on soil properties. (A) Soil organic matter content. (B) Alkali-hydrolyzable nitrogen content. (C) Available phosphorus content. (D) Available potassium content.

The organic matter and alkali-hydrolyzable nitrogen contents of the soils treated with IBP, BP, and FA were significantly greater than those of the control CK at 18 days and 36 days posttransplantation (Fig. 2), indicating that the application of fulvic acid and *B. paralicheniformis* ferment increased the fertility and nitrogen supply levels of the soil and that the application of *B. paralicheniformis* ferment had better effect. The available phosphorus and potassium contents of FA treatment were significantly (*P* < 0.01) greater than control CK at 18 days and 36 days posttransplantation. The available potassium content of IBP and BP treatments was significantly greater than control CK at 36 days posttransplantation. Application of *B. paralicheniformis* 285-3 ferment and fulvic acid powder both improved soil physicochemical properties.

### Microbial community changed after application of fulvic acid and *B. paralicheniformis* ferment.

At 18 days posttransplantation, the number of bacterial operational taxonomic units (OTUs) unique to IBP, BP, FA, and CK treatments was 408, 344, 375, and 315, respectively, and the common number of OTUs was 5,096 (see Fig. S1 in the supplemental material). The number of bacterial species observed in IBP, BP, and FA treatments was greater than that in control CK. Application of fulvic acid and *B. paralicheniformis* ferment increased bacterial species at 18 days posttransplantation.

At 36 days posttransplantation, the number of species (i.e., OTU number) observed in the IBP, BP, FA, and CK treatments was 2,621, 2,433, 2,453, and 1,976, respectively. Compared with the 18 days posttransplantation results, the common OTU number of four treatments was significantly reduced; the unique OTU number of IBP, BP, and FA treatments all increased; and the unique OTU number of CK decreased (see Fig. S2 in the supplemental material). The number of bacterial species observed in IBP, BP, and FA treatments was higher than that in control CK. The IBP group contained the greatest number of OTUs.

The alpha diversity can reflect the microbial community’s diversity and richness. There was no significant difference between the alpha diversity of IBP, BP, and FA treatments and the control CK at 18 days posttransplantation (see Table S1 in the supplemental material). The alpha diversity of IBP, BP, and FA treatments was different from the control at 36 days posttransplantation (Table 1). The higher the community diversity and the more uniform the species distribution, the greater the Shannon index. The Shannon indices of IBP and FA treatments were significantly (*P* < 0.05) greater than that of the control CK. The overall Shannon index at 18 days posttransplantation was greater than that at 36 days posttransplantation. Simpson indices characterize the diversity and evenness of species distribution within the community, and species were evenly distributed within each treatment community. The Chao1 index was used to estimate the total number of species included in the community, and the total number of species in IBP, BP, and FA treatments were significantly (*P* < 0.05) greater than in the control CK. The total number of species of all treatments at 18 days posttransplantation was greater than those at 36 days posttransplantation. The ACE index was used to estimate the OTU numbers in the community. ACE indices of IBP, BP, and FA treatments were significantly (*P* < 0.05) greater than those of the control CK at 36 days posttransplantation. The ACE index of all treatments at 36 days decreased compared to that at 18 days posttransplantation. Application of fulvic acid and *B. paralicheniformis* ferment increased microbial diversity at 36 days posttransplantation, and heat-treated *B. paralicheniformis* ferment had the better effect.

**TABLE 1:** Statistics analysis of alpha diversity of bacteria at 36 days posttransplantation^*a*^

Treatment	OTU no.	Shannon value	Simpson value	Chao1 value	ACE value
IBP36	2,621.8 ± 122.8 a	9.17 ± 0.27 a	0.990 ± 0.006 a	2,900.1 ± 129.9 a	2,971.5 ± 134.7 a
BP36	2,433.8 ± 214.0 a	8.82 ± 0.34 bc	0.987 ± 0.006 a	2,726.2 ± 233.5 a	2,779.9 ± 197.8 b
FA36	2,453.4 ± 176.3 a	8.93 ± 0.44 ab	0.990 ± 0.005 a	2,718.6 ± 174.3 a	2,767.1 ± 168.0 b
CK36	1,976.9 ± 225.4 b	8.57 ± 0.16 c	0.991 ± 0.002 a	2,403.5 ± 284.1 b	2,441.0 ± 247.6 c

a The different letters in the same column represent significant difference between treatments. IBP36, soil treated with inactive Bacillus paralicheniformis ferment at 36 days posttransplantation; BP36, soil treated with *B. paralicheniformis* ferment at 36 days posttransplantation; FA36, soil treated with fulvic acid powder at 36 days posttransplantation; CK36, untreated soil as control at 36 days posttransplantation.

**FIG 3:**
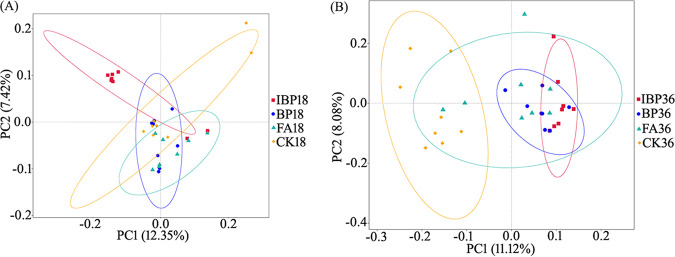
PCoA of soil bacterial community at 18 days posttransplantation (A) and at 36 days posttransplantation (B).

The difference in the microbial community composition of the four treated soils was analyzed by principal-coordinate analysis (PCoA), and the distance between the treatment samples represented the degree of difference. At 18 days posttransplantation, the samples of IBP treatment were clustered, concentrated, and separated from CK samples. At 36 days posttransplantation, IBP, BP, and FA communities were all quite different from control CK, indicating that both fulvic acid and *B. paralicheniformis* ferment application changed soil bacterial community structures (Fig. 3). Based on multi-response permutation procedures (MRPP) analysis, microbial communities of IBP, BP, and FA treatments were all significantly different from the control CK both at 18 days and 36 days posttransplantation (Table 2). Analysis of similarity (ANOSIM), multivariate analysis of variance (Adonis), and analysis of molecular variance (AMOVA) all showed that BP, IBP, and FA communities were significantly (*P* < 0.05) different from CK at 36 days posttransplantation. Community difference between fulvic acid and *B. paralicheniformis* ferment-treated soils and control soil at 36 days was greater than that at 18 days posttransplantation.

**TABLE 2:** MRPP analysis of microbial community

Group	18 days posttransplantation^*a*^		36 days posttransplantation^*a*^	
	*A* value	Significance	*A* value	Significance
IBP-CK	0.097	0.005	0.11	0.001
BP-CK	0.077	0.001	0.104	0.001
FA-CK	0.039	0.015	0.072	0.001

a An *A* value greater than 0 indicates that differences between groups are greater than within-group differences. A significance value less than 0.05 indicates a significant difference.

**FIG 4:**
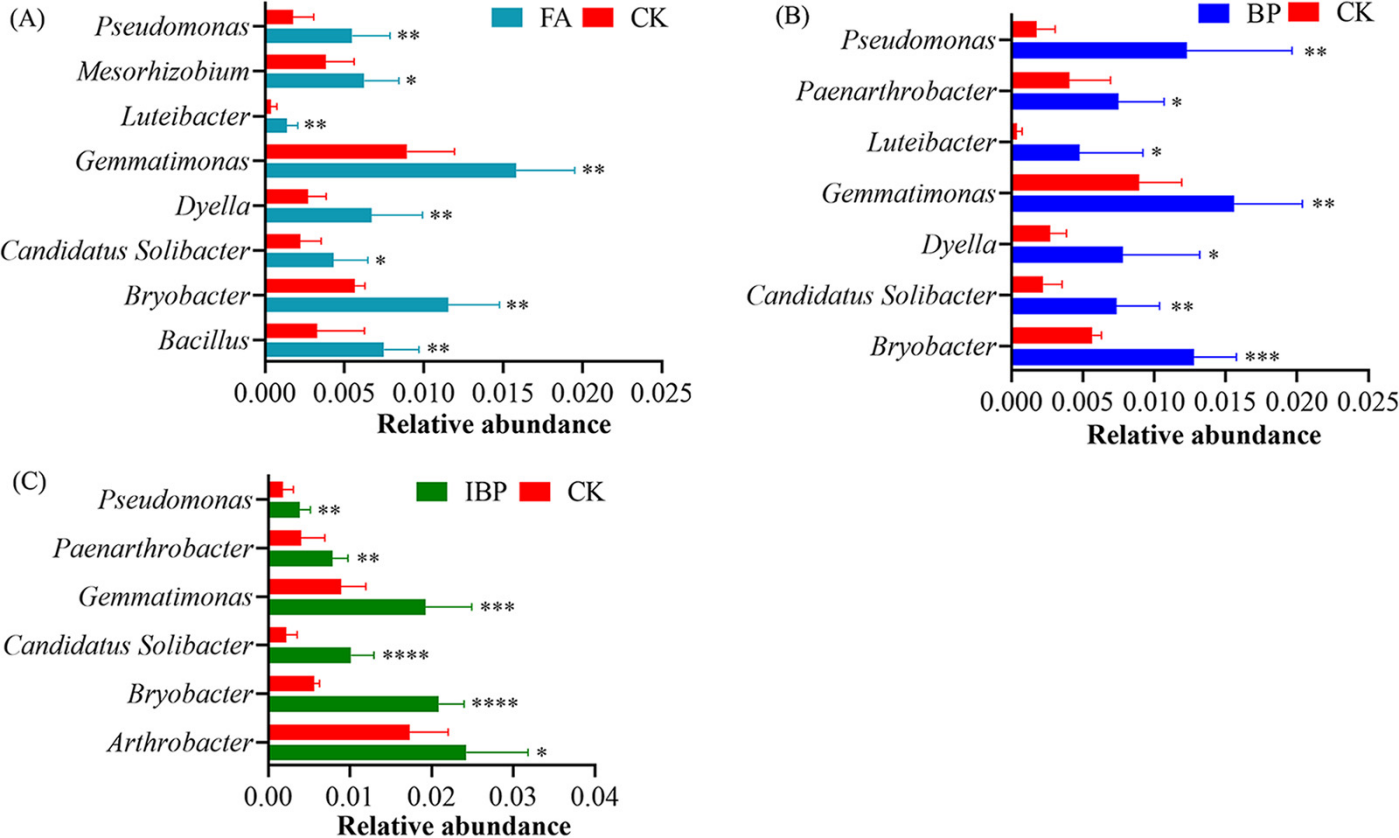
Comparing relative abundance of bacterial genera in four groups at 36 days posttransplantation by *t* test. (A) Genera difference between FA treatment and control CK. (B) Genera difference between BP treatment and control CK. (C) Genera difference between IBP treatment and control CK.

At 36 days posttransplantation, BP treatment enriched beneficial bacteria including *Bryobacter*, “*Candidatus* Solibacter,” *Dyella*, *Ellin6067*, *Gemmatimonas*, *Luteibacter*, *Paenarthrobacter*, and *Pseudomonas* (Fig. 4). FA treatment enriched beneficial bacteria including *Bacillus*, *Bryobacter*, “*Candidatus* Solibacter,” *Dyella*, *Gemmatimonas*, *Luteibacter*, *Mesorhizobium*, and *Pseudomonas*. IBP treatment enriched beneficial bacteria, including *Arthrobacter*, *Bryobacter*, “*Candidatus* Solibacter,” *Gemmatimonas*, *Paenarthrobacter*, and *Pseudomonas*. Application of fulvic acid and *B. paralicheniformis* ferment enriched plant beneficial bacteria.

### Soil microbial network changed after application of fulvic acid and Bacillus paralicheniformis ferment at 36 days posttransplantation.

**FIG 5:**
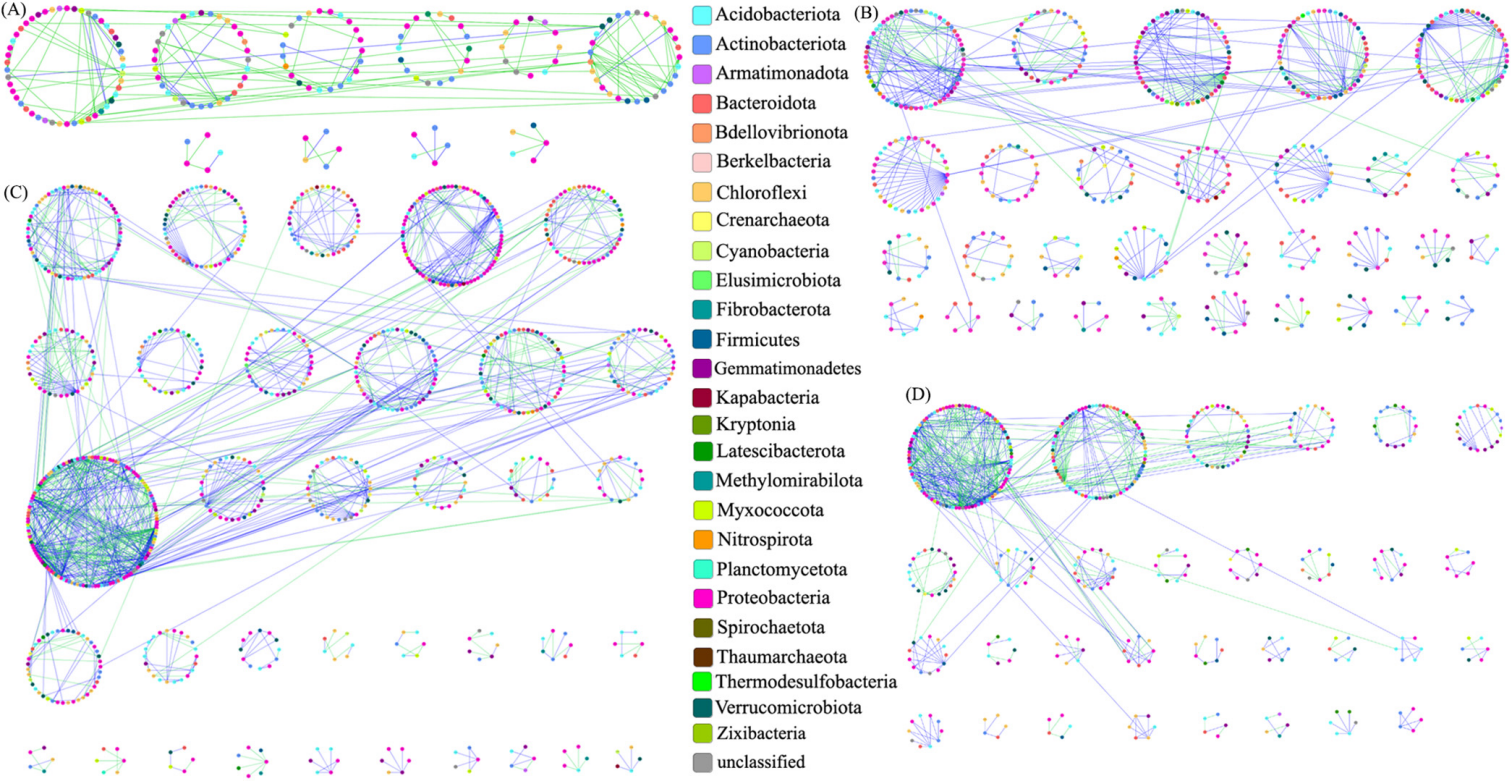
Overview of CK network (A), FA network (B), IBP network (C), and BP network (D) at 36 days posttransplantation. Different colors of the nodes belong to different bacterial phyla. Green lines and blue lines indicate negative and positive interactions between different nodes, respectively.

At 36 days posttransplantation when compared to those of the control, the microbial networks of FA, BP, and IBP treatments had a greater number of nodes, closer microbial connection, and higher complexity; that is, each treatment (FA, BP, IBP) increased the size and complexity of soil microbial network (Fig. 5). The close similar threshold (*S_t_*) value was obtained for the FA (0.94), BP (0.94), IBP (0.93), and CK (0.95) networks. Applying these thresholds, four networks were constructed for the CK-, FA-, BP-, and IBP-treated soils, respectively (Fig. 5). The CK, FA, BP, and IBP networks consisted of 339, 885, 810, and 1,265 nodes (OTUs) and 307, 913, 1,029, and 1,875 edges (connections), respectively, indicating that the FA, BP, and IBP networks had a larger size with more nodes and links than the control CK network. IBP network had the largest size among the four networks. After application of fulvic acid and *B. paralicheniformis* ferment, microbial network had far more complicated interactions in terms of network size, connectivity, and clustering coefficient when compared with the control CK network (Table 3). In the FA, BP, and IBP networks, 66.8% (610/912), 53.7% (553/1,028), and 57.5% (1,078/1,874) of the nodes had positive interactions with others, indicating that the microbial community under fulvic acid or *B. paralicheniformis* ferment-treated soils formed a close organization via synergistic interactions among different species. In the control CK network, 70.9% (217/306) of the nodes had negative interactions, suggesting that there were more competition and antagonism among different bacterial species in the untreated soil.

**TABLE 3:** Topological properties of the microbial network and their associated random networks at 36 days posttransplantation^*a*^

Network	Network size	Empirical network						Random network		
		*S_t_*	*R^2^*	avgK	avgGD	avgCC	M (no. of modules)	avgGD	avgCC	avgM
CK	339	0.95	0.98	1.81	3.85	0.08	0.88 (95)	6.44 ± 0.36	0.003 ± 0.003	0.86 ± 0.01
FA	885	0.94	0.93	2.06	9.59	0.06	0.90 (170)	6.60 ± 0.18	0.002 ± 0.002	0.83 ± 0.006
BP	810	0.94	0.93	2.54	5.44	0.12	0.80 (172)	5.07 ± 0.08	0.009 ± 0.002	0.70 ± 0.005
IBP	1,265	0.93	0.89	2.96	9.62	0.12	0.83 (160)	5.27 ± 0.05	0.006 ± 0.001	0.65 ± 0.003

a The random networks are generated by rewiring all of the links of a phylogenetic molecular ecological network with the identical numbers of nodes and links to the corresponding empirical phylogenetic molecular ecological network. *S_t_*, similar thresholds; avgK, average connectivity; avgGD, average geodesic distance; avgCC, average clustering coefficient; M, modularity; avgM, average modularity; CK, control group; FA, fulvic acid treatment; BP, fermentation broth of Bacillus paralicheniformis 285-3; IBP, the heated fermentation broth of Bacillus paralicheniformis 285-3.

Connectivity represents the number of links between a node and other nodes. Higher average connectivity (avgK) means a more complex network. The avgK of FA (2.06), BP (2.54), and IBP (2.96) networks was higher than that of the control CK network (1.81), suggesting that the FA, BP, and IBP networks were more complex than the CK network. All curves of network connectivity distribution were fitted well with the power-law model (*R^2^* values varied from 0.89 to 0.98), indicative of scale-free networks. Geodesic distance represents the shortest path between two nodes. The average network distance (avgGD) between all pairs of nodes was 3.85, 9.59, 5.44, and 9.62 edges for the CK, FA, BP, and IBP networks, respectively, suggesting that OTUs in the FA, BP, and IBP networks were more highly connected with each other when compared with the control CK network. Clustering coefficient describes how well a node is connected with its neighbors. Average clustering coefficient (avgCC) is used to measure the extent of module structure present in a network. The avgCC of BP (0.12) and IBP (0.12) was higher than that of CK (0.08), indicating that nodes in the BP and IBP network tended to better cluster together when compared with those in the CK network. Modularity is used to demonstrate a network that can be naturally divided into communities or modules. Each module in the microbial network is considered as a functional unit that consists of several elementary bacteria and performs an identifiable task (11). Modularity (M) values of the FA, BP, IBP, and CK networks were all higher than the threshold value for modular structure (0.4), suggesting that these four networks were all modular (12). The geodesic distance, clustering coefficient, and modularity of these four phylogenetic molecular ecological networks (pMENs) were significantly different from those of the corresponding random networks with the same network size and number of links, indicating that these microbial networks showed typical small-world characteristics. The geodesic distance, clustering coefficient, and modularity of these pMENs were significantly different between control and the three treatments (i.e., FA, BP, IBP). These results indicated that the network composition and structure of fulvic acid and *B. paralicheniformis* ferment-treated soils differed from the untreated soil. Application of fulvic acid and *B. paralicheniformis* ferment shifted microbial network structure. The IBP network had higher connectivity and geodesic distance than the FA and BP networks, indicating that application of heat-treated *B. paralicheniformis* ferment increased complexity of network compared to fulvic acid powder and *B. paralicheniformis* ferment application. The number of unique nodes in IBP, BP, and FA treatments was greater than control CK. The IBP network had the most unique nodes followed by the FA network (see Fig. S3 in the supplemental material).

### Comparing the keystone microorganisms of four networks.

**FIG 6:**
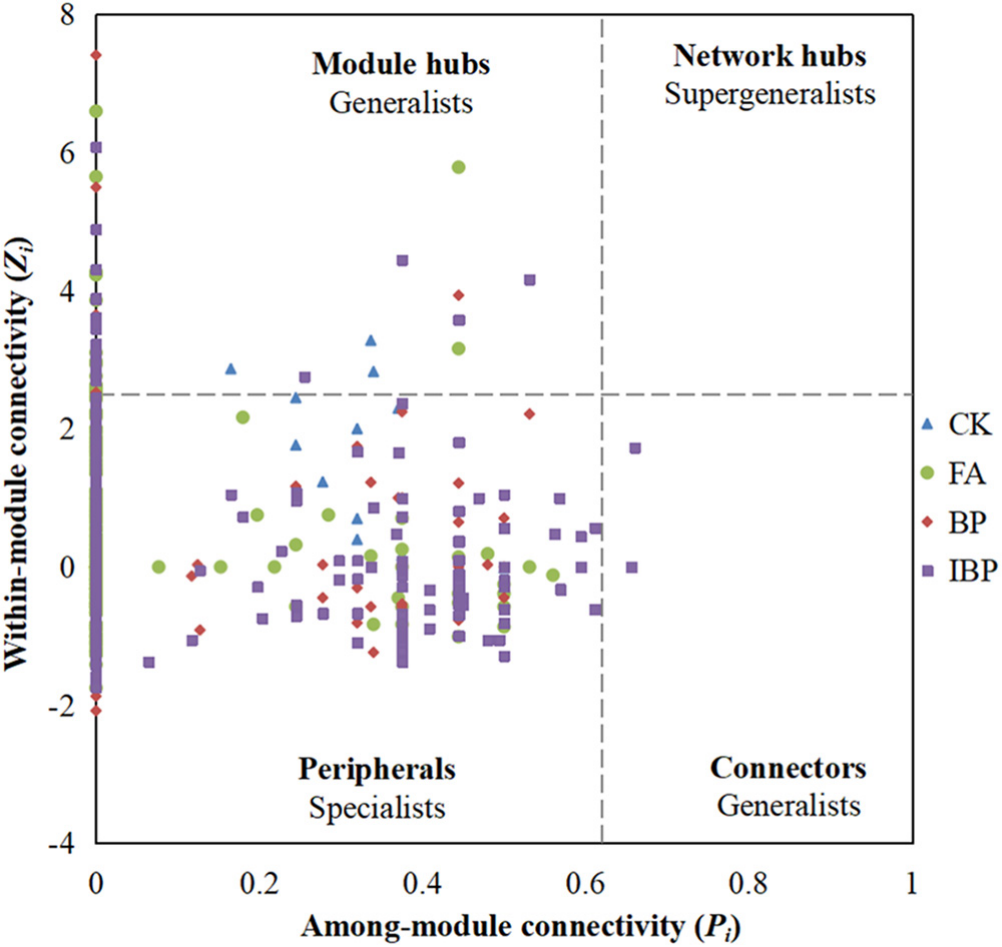
*Zi*-*Pi* plot indicates the distribution of operational taxonomic units (OTUs) based on their topological roles. Each symbol represents an OTU in the control (CK) network (blue triangle), FA network (green circle), BP network (red diamond), and IBP network (purple square). The threshold values of *Zi* and *Pi* for categorizing OTUs are 2.5 and 0.62, respectively.

Topological roles of nodes were shown in Fig. 6. According to the threshold values of within-module connectivity (*Zi*) (2.5) and among-module connectivity (*Pi*) (0.62), all nodes were divided into peripherals (specialists), connectors (generalists), and module hubs (generalists), but no nodes fell into network hubs (supergeneralists) (13). Most of the nodes (99.1%, 98.2%, 98.8%, and 97.4% for the CK, FA, BP, and IBP networks, respectively) were peripherals. Totals of 93.8%, 92.5%, 91.5%, and 89.4% of peripherals in the CK, FA, BP, and IBP networks had no links to other nodes outside of their own modules (*Pi *= 0). Totals of 3, 16, 10, and 33 keystone microorganisms were identified in the CK, FA, BP, and IBP networks, respectively (Table 4). Application of fulvic acid and *B. paralicheniformis* ferment increased the number of keystone microorganisms.

**TABLE 4:** The keystone microorganisms of microbial network

Network	Keystone microorganism	No. module	Phylum	Genus	Correlation with disease severity index^*a*^
CK	OTU74	1	*Acidobacteriota*		**0.95 (0.05)**
	OTU288	0	*Bdellovibrionota*	*Oligoflexus*	0.63 (0.37)
	OTU569	0	*Actinobacteriota*	*Atopobium*	0.62 (0.38)
FA	OTU13	5	*Proteobacteria*		0.09 (0.91)
	OTU16	24	*Cyanobacteria*		0.73 (0.27)
	OTU43	0	*Proteobacteria*	*Mesorhizobium*	−0.84 (0.16)
	OTU73	2	*Proteobacteria*		−0.74 (0.27)
	OTU401	16	*Gemmatimonadetes*		−0.88 (0.12)
	OTU446	4	*Actinobacteriota*	*Streptomyces*	0.07 (0.94)
	OTU480	15	*Acidobacteriota*	*Bryobacter*	−0.10 (0.90)
	OTU763	1	*Firmicutes*	*Bacillus*	**−0.99 (0.02)**
	OTU820	8	*Bacteroidota*	*Edaphobaculum*	0.11 (0.89)
	OTU1009	7	*Proteobacteria*		−0.75 (0.25)
	OTU1036	18	*Proteobacteria*		−0.40 (0.6)
	OTU1458	11	*Acidobacteriota*	“*Candidatus* Solibacter”	−0.23 (0.77)
	OTU2697	9	*Acidobacteriota*		−0.29 (0.72)
	OTU3541	25	*Proteobacteria*	*Thermomonas*	0.20 (0.80)
	OTU5282	12	*Actinobacteriota*		0.74 (0.27)
	OTU7318	3	*Proteobacteria*	*Sphingomonas*	**−0.95 (0.049)**
BP	OTU94	6	*Crenarchaeota*		−0.22 (0.78)
	OTU185	2	*Gemmatimonadetes*	*Gemmatimonas*	−0.32 (0.68)
	OTU186	3	*Chloroflexi*		−0.01 (0.99)
	OTU203	1	*Cyanobacteria*		0.15 (0.85)
	OTU446	0	*Actinobacteriota*	*Streptomyces*	0.07 (0.94)
	OTU489	5	*Proteobacteria*	*Polycyclovorans*	0.63 (0.37)
	OTU869	0	*Proteobacteria*		0.11 (0.89)
	OTU1287	14	*Firmicutes*	*Bacillus*	0.16 (0.84)
	OTU1332	0	*Actinobacteriota*		0.54 (0.46)
	OTU1512	1	*Acidobacteriota*	“*Candidatus* Koribacter”	0.23 (0.77)
IBP	OTU35	3	*Proteobacteria*	*Devosia*	0.42 (0.58)
	OTU37	5	*Proteobacteria*		0.47 (0.53)
	OTU71	11	*Proteobacteria*		−0.57 (0.43)
	OTU136	15	*Gemmatimonadetes*		0.06 (0.94)
	OTU260	11	*Proteobacteria*	*Dongia*	**−0.98 (0.03)**
	OTU291	1	*Actinobacteriota*	*Humibacter*	−0.04 (0.96)
	OTU352	13	*Proteobacteria*	*Ramlibacter*	0.42 (0.58)
	OTU373	11	*Proteobacteria*		−0.44 (0.56)
	OTU394	9	*Verrucomicrobiota*		0.18 (0.83)
	OTU400	11	*Bacteroidota*		0.44 (0.56)
	OTU443	0	*Firmicutes*	*Bacillus*	−0.19 (0.81)
	OTU490	8	*Proteobacteria*	*Rhodanobacter*	−0.23 (0.77)
	OTU494	6	*Proteobacteria*	*Ellin6067*	0.64 (0.36)
	OTU556	17	*Bacteroidota*	*Pricia*	0.01 (0.99)
	OTU759	14	*Bacteroidota*		−0.52 (0.48)
	OTU881	12	*Actinobacteriota*	*Paenarthrobacter*	0.60 (0.40)
	OTU968	11	*Chloroflexi*		−0.19 (0.81)
	OTU1364	4	*Proteobacteria*		0.05 (0.95)
	OTU1428	1	*Proteobacteria*	*Mesorhizobium*	0.51 (0.49)
	OTU1537	10	*Methylomirabilota*		0.01 (0.99)
	OTU1545	3	*Kapabacteria*		0.23 (0.77)
	OTU2819	11	*Proteobacteria*		0.19 (0.81)
	OTU3275	3	*Proteobacteria*	*Rhodanobacter*	−0.71 (0.29)
	OTU3657	3	*Proteobacteria*		−0.81 (0.19)
	OTU4207	8	*Acidobacteriota*		−0.33 (0.67)
	OTU4362	16	*Chloroflexi*		0.61 (0.39)
	OTU4557	11	*Proteobacteria*	*Sphingomonas*	−0.25 (0.75)
	OTU4754	11	*Proteobacteria*	*Sphingomonas*	0.74 (0.26)
	OTU5164	2	*Actinobacteriota*	*Microbacterium*	−0.73 (0.27)
	OTU5196	0	*Acidobacteriota*	*Bryobacter*	−0.03 (0.98)
	OTU5443	6	*Proteobacteria*	*Chujaibacter*	−0.53 (0.47)
	OTU7120	2	*Proteobacteria*	*Chujaibacter*	−0.91 (0.09)
	OTU7262	9	*Actinobacteriota*	*Catellatospora*	−0.78 (0.22)

a Number in brackets represent the *P* value. Bold numbers represent significant correlation.

In the CK network, 3 generalists (3 module hubs) belonged to *Acidobacteriota*, *Actinobacteriota*, and *Bdellovibrionota*, respectively. OTU288 and OTU569 were respectively closely related to *Oligoflexus* and *Atopobium*. OTU74 belonging to *Acidobacteriota* was positively correlated to disease severity index (*r *= 0.95; *P = *0.05). In the FA network, 16 generalists (16 module hubs) belonged to *Acidobacteriota*, *Actinobacteriota*, *Bacteroidota*, *Cyanobacteria*, *Firmicutes*, *Gemmatimonadetes*, and *Proteobacteria*, respectively. OTU446 and OTU763 were respectively closely related to *Streptomyces* and *Bacillus*. OTU763 was negatively correlated to disease severity index (*r* = −0.99; *P = *0.02). OTU43 was closely related to rhizobium *Mesorhizobium*. OTU480 was closely related to *Bryobacter*. OTU1458 was closely related to “*Candidatus* Solibacter.” OTU3541 was closely related to denitrifying bacterium *Thermomonas*. OTU7318 was closely related to *Sphingomonas* and negatively correlated to disease severity index (*r* = −0.95; *P = *0.049). In the BP network, 10 generalists belonged to *Acidobacteriota*, *Actinobacteriota*, *Chloroflexi*, *Crenarchaeota*, *Cyanobacteria*, *Firmicutes*, *Gemmatimonadetes*, and *Proteobacteria*, respectively. OTU185 was closely related to phototrophic *Gemmatimonas*. OTU1512 was closely related to “*Candidatus* Koribacter.” OTU446 was closely related to *Streptomyces*. OTU1287 was closely related to *Bacillus*. In the IBP network, 33 generalists (30 module hubs, 3 connectors) belonged to *Acidobacteriota*, *Actinobacteriota*, *Bacteroidota*, *Chloroflexi*, *Firmicutes*, *Gemmatimonadetes*, *Kapabacteria*, *Methylomirabilota*, *Proteobacteria*, and *Verrucomicrobiota*, respectively. OTU35 was closely related to *Devosia*. OTU260 was closely related to *Dongia* and negatively correlated to disease severity index (*r* = −0.98; *P = *0.03). OTU352 was closely related to plant-beneficial *Ramlibacter*. OTU490 was closely related to beneficial bacteria *Rhodanobacter*. OTU494 was closely related to nitrosifying bacteria *Ellin6067*. OTU881 was closely related to *Paenarthrobacter*. OTU1428 was closely related to rhizobium *Mesorhizobium*. OTU3275 was closely related to beneficial bacteria *Rhodanobacter*. OTU4557 and OTU4754 were closely related to *Sphingomonas*. OTU5164 was closely related to *Microbacterium*. OTU5196 was closely related to plant growth promoting rhizobacteria *Bryobacter*. OTU5443 and OTU7120 were closely related to *Chujaibacter*. OTU7262 was closely related to *Catellatospora*. It could be seen that keystone microorganisms were definitely different between the four groups. Application of heat-treated *B. paralicheniformis* ferment enhanced the microbial network complexity and increased the number of keystone microorganisms. In FA, BP, and IBP networks, keystone microorganisms included some plant-beneficial taxa like *Bacillus*, *Ramlibacter*, *Sphingomonas*, *Catellatospora*, and *Mesorhizobium*, which might improve soil health, inhibit bacterial wilt disease, and indirectly benefit the plant growth.

## DISCUSSION

### Application of fulvic acid and *B. paralicheniformis* ferment improved soil fertility and inhibited bacterial wilt disease.

Fulvic acid is an organic aromatic substance with high activity and is a biostimulator. In the present study, it was first found that fulvic acid powder and the fulvic acid fermented by *B. paralicheniformis* could inhibit bacterial wilt disease (Fig. 1). Biocontrol efficacy of fulvic acid (99.1%) was better than the chitosan and chitosan-derived nanoparticles reported previously (62%) (14) and biological control agents (49.3%) (15). In our previous study, fulvic acid did not exhibit antibacterial activity *in vitro* and could not directly inhibit the growth of pathogen R. solanacearum causing the widespread disease known as bacterial wilt. Fulvic acid and γ-PGA produced by *B. paralicheniformis* might induce tobacco plant defense responses that indirectly inhibited bacterial wilt disease. It has been reported previously that fulvic acid induces resistance to Botrytis cinerea through the activation of the phenylpropanoid pathway (9). Xu et al. (16) found that application of γ-PGA increased the fresh weight, chlorophyll content, proline content, and antioxidant enzyme activity of rape seedlings.

Application of fulvic acid and *B. paralicheniformis* ferment increased soil organic matter (Fig. 2). Zhang et al. (17) found that exogenous fulvic acid (900 kg ha^−1^) applications, which derived from the biological fermentation of maize straw, increased soil organic carbon by 26.93%. FA (fulvic acid, 150 kg ha^−1^), which was derived from the yeast molasses fermentation, increased soil organic carbon by 62.05%. Different sources of fulvic acid have different effects on improving soil organic matter. Fulvic acid extracted from the molasses fermented by yeast had a better effect on improving soil carbon content than that extracted from maize straw ferment. The BP and IBP treatments increased soil organic carbon by 64.96% and 73.38%, respectively. Fulvic acid and γ-PGA produced by *B. paralicheniformis* showed additive effect on improving soil carbon and nitrogen nutrients. Fulvic acid application also increased soil N, P, and K contents. At 36 days posttransplantation, application of fulvic acid powder increased available phosphorus in soil by 185.69% compared with that of soil untreated with fulvic acid, which had better effect than the fulvic acid extracted from paper mill effluent used in a previous study (47.5 to 57.5% increase of phosphorus) (18). At 36 days posttransplantation, IBP, BP, and FA treatments increased alkali-hydrolyzable nitrogen in soil by 101.25%, 127.07%, and 90.43%, respectively. Few studies reported the effect of fulvic acid on soil alkali-hydrolyzable nitrogen content. At 36 days posttransplantation, IBP, BP, and FA treatments increased available potassium in soil by 53.19%, 43.27%, and 51.49% compared with that of the untreated soil, which showed better effect than potassium fulvic acid used in previous study (4.23% increase in soil available potassium) (19). Fulvic acid could provide potassium necessary for the growth of crops. However, Wang et al. (18) found that application of fulvic acid extracted from paper mill effluent had no remarkable effects on soil pH, inorganic nitrogen, available potassium, and organic matter. The effects of fulvic acid from different sources on soil nutrition varied, and the fulvic acid extracted from yeast fermentation broth had a better effect of improving soil fertility. Effect of fulvic acid and *B. paralicheniformis* ferment on soil properties at 18 days differed from that at 36 days posttransplantation, and it was supposed that fulvic acid and γ-PGA in soil was absorbed by plants as the plant grew.

### Application of fulvic acid and *B. paralicheniformis* ferment changed soil bacterial community diversity, structure, and composition.

The effect of fulvic acid and *B. paralicheniformis* ferment on microbiota became more obvious at 36 days than at 18 days posttransplantation. So, the following analysis was focused on microbiota of 36 days. Application of fulvic acid and *B. paralicheniformis* ferment increased bacterial community diversity and numbers in soil (Table 1). Fulvic acid extracted from yeast molasses fermentation was rich in various metabolites, nitrogen, and carbon sources (i.e., amino acids, proteins, organic acids) that might provide nutrients for bacterial growth in rhizosphere soil, thus increasing bacterial diversity and quantity. For the BP and IBP treatments, fulvic acid was fermented with *B. paralicheniformis* 285-3. The γ-PGA produced by *B. paralicheniformis* 285-3 provided a nitrogen source for soil bacteria growth and possibly enhanced bacterial stress resistance. Microbiota in IBP treatment had greater Shannon and ACE indices than in BP and FA treatments. Heat-treated γ-PGA had a smaller molecular weight than unheated γ-PGA, and thus was more easily absorbed and utilized by microorganisms, and was easier to improve plant immune resistance (Fig. 1) based on a previous study (20). Accordingly microbial community structures of IBP, BP, and FA treatments differed from the control CK (Fig. 3).

Application of fulvic acid and *B. paralicheniformis* ferment increased the abundances of plant beneficial bacteria in soil, which had the potential to promote plant growth. Relative abundance of *Dyella* in BP and FA treatments was greater than in control CK (Fig. 4). *Dyella* has shown various plant growth-promoting activities and the potential as a biological control agent (21). Relative abundance of *Luteibacter* in BP and FA treatments were greater than in control CK. Guglielmetti et al. (22) found that *Luteibacter* had the ability to produce molecules able to chelate ferric ions and solubilize monocalcium phosphate, produce indole acetic acid, and promote root development. Relative abundance of *Arthrobacter* in IBP was greater than in control CK. Krishnan et al. (23) found that *Arthrobacter* possessed plant growth beneficial traits such as positive growth on 1-aminocyclopropane-1-carboxylic acid and production of indole acetic acid and siderophore. The relative abundance of *Caulobacter* in BP treatment was greater than in control CK. Berrios (24) found that several *Caulobacter* strains harbored the potential to enhance plant biomass. The relative abundances of Bacillus aryabhattai, Bacillus sporothermodurans, and *Gemmatimonas* in the FA treatment were greater than in control CK. Deng et al. (25) reported that Bacillus aryabhattai had phosphate-solubilizing and nitrogen-fixing functions that promote plant growth. Osaki et al. (26) found that *B. sporothermodurans* produced volatile compounds that showed inhibitory activity against pathogenic fungi. Mujakić et al. (27) reported that *Gemmatimonas* had the capacity for anoxygenic photosynthesis. Plant beneficial bacteria enriched in fulvic acid and *B. paralicheniformis* ferment-treated soil might improve soil fertility and inhibit bacterial wilt disease.

### Application of fulvic acid and *B. paralicheniformis* ferment increased soil bacterial network complexity and stability.

The microbial networks of fulvic acid and *B. paralicheniformis* ferment-treated soils had more complicated interactions than the control CK network at 36 days posttransplantation (Table 3). Qi et al. (28) reported that microbial network of healthy soil was more complex than susceptible soil network, which was conducive to enhancing soil health and disease suppression. The increase in bacterial network complexity after application of fulvic acid and *B. paralicheniformis* ferment possibly enhanced soil health and inhibited pathogen R. solanacearum infection. The IBP network was the most complex and had the largest size among all treatments. It was supposed that fulvic acid and γ-PGA in fermentation broth had a synergistic action to enhance the stability of the microbial network.

Compared with untreated soil, more synergistic interactions among bacterial species were found in fulvic acid and *B. paralicheniformis* ferment-treated soils (Fig. 5). The synergy between microorganisms can promote microbial growth, complete more complex metabolic processes, produce new metabolites, and occupy more ecological niches. For example, Corrêa et al. (29) observed the synergy between the enzymes produced by Trichoderma reesei RUT-C30 and Penicillium oxalicum to break down the cellulose fraction of sugarcane straw. It was speculated that the microbiota of soil treated with fulvic acid and *B. paralicheniformis* ferment was more stable and could resist the infection of foreign phytopathogens compared to untreated soil. Bacterial species in the untreated soil had more competition and antagonism. The antagonism and competition among microorganisms mean that different microorganisms compete for space and nutrients, which inhibit the growth of some microorganisms, decrease the microbial species, and reduce microbial diversity. The microbial community of untreated soil was loose and unstable and susceptible to phytopathogen. Based on the theory of Cray et al. (30), pathogen R. solanacearum might act as the microbial weed species and struggle to dominate in the control soil. Why could the alien species R. solanacearum invade native communities and become widespread in untreated soil? It was speculated that low microbial community diversity and unstable network structure of untreated soil favored the pathogen infection; while an increase of microbial diversity and a more stable network of fulvic acid and *B. paralicheniformis* ferment treated soil was conducive to resist pathogen infection.

### Keystone microorganisms changed after application of fulvic acid and *B. paralicheniformis* ferment.

The keystone microorganisms of FA, BP, and IBP networks differed from those of the control network (Table 4). Some keystone microorganisms in fulvic acid and *B. paralicheniformis* ferment-treated soils were potential plant beneficial bacteria, which might promote the growth of plants, produced antimicrobial substances, and participated in the soil nutrient cycle. It could be speculated from previous reports that these beneficial keystone microorganisms had important roles in improving soil quality and disease suppression. For instance, *Streptomyces* and *Bacillus* were the keystone microorganisms of FA and BP networks. *Bacillus* was the keystone microorganism of IBP network. *Bacillus* and *Streptomyces* have been popularly used to control plant diseases, such as bacterial wilt disease (31, 32). Keystone microorganism *Bacillus* (OTU763) was negatively correlated to disease severity index, indicating *Bacillus* played a key role in inhibiting R. solanacearum. Ward et al. (33) found that “*Candidatus* Koribacter” (related to OTU1512) could use complex substrates (e.g., chitin, hemicelluloses, cellulose, pectin, starch, and xylan) and produced antimicrobial compounds. Huo et al. (34) reported that *Rhodanobacter* (related to OTU490) was plant growth-promoting rhizobacteria and showed antagonistic activity against the root rot pathogen. Some of the species from the *Sphingomonas* (related to OTU4557 and OTU4754) have been found to improve plant growth during stress conditions and possess antimicrobial activity (35, 36). *Microbacterium* (related to OTU5164) is characterized by high antimicrobial activity and produces the potent antibacterial lanthipeptide microvionin (37, 38). It was speculated that antibacterial substances produced by keystone microorganisms could inhibit the growth of pathogens in the soil and occupy more ecological niches. Rawat et al. (39) reported that “*Candidatus* Solibacter” (related to OTU1458) decomposed organic carbon and carbohydrate in the soil, thus improving soil quality. The *Mesorhizobium* (related to OTU43), *Bryobacter* (related to OTU480), *Devosia* (related to OTU35), *Paenarthrobacter* (related to OTU881), and *Catellatospora* (related to OTU7262) have shown plant growth-promoting activities (i.e., phosphorous, potassium solubilization, nitrogen-fixing activity, and production of siderophore, indole acetic acid, and ansamacrolactams) (40–45). Beneficial keystone microorganisms possibly promoted plant growth, inhibited bacterial wilt disease involved in the biogeochemical C and N cycles of soil, and had a beneficial ecological function of enhancing soil fertility. The application of fulvic acid and *B. paralicheniformis* ferment shifted the bacterial community and network; keystone microorganisms included biocontrol bacteria (i.e., *Bacillus*, *Rhodanobacter*, *Streptomyces*), plant growth-promoting rhizobacteria (i.e., *Bryobacter*, *Catellatospora*, *Devosia*, *Paenarthrobacter*), and soil nutrient cycling-affecting bacteria (i.e., *Bryobacter*, *Candidatus Solibacter*, *Mesorhizobium*, *Thermomonas*), which were valuable for the promotion of plant growth and health. The number of keystone microorganisms in the untreated soil was small, and no beneficial bacteria were found. The keystone microorganism OTU74 belonging to *Acidobacteriota* was positively correlated to disease severity index, and acidophilic *Acidobacteria* might easily live and grow in the acidic soil. Zhang et al. (46) reported that bacterial wilt was accompanied by soil acidification, and the abundance of R. solanacearum increased when the pH of soil reduced. It was speculated that *Acidobacteria* might act as a helper of pathogen R. solanacearum. This speculation will be studied in the future.

## MATERIALS AND METHODS

### Plot experiment.

The plot experiment was conducted in tobacco fields located in Enshi State, Xuanen County (29°95′N, 109°38′E), with a subtropical humid climate, a mean annual temperature of 16°C, and mean annual precipitation of 1,400 mm. Soil type was yellow-brown soil (classified as Alfisols). Tobacco (Nicotiana tabacum L.) variety Yunyan 87 was planted in this area for more than 10 years. Each plant was separated by 0.55 m. Four leaf-stage seedlings were transplanted to the field in May 2021.

The trial used a randomized block design with a total of four treatments including control (CK), fulvic acid powder (FA), Bacillus paralicheniformis ferment (BP), and heat-treated Bacillus paralicheniformis ferment (IBP) (Table 5). Eight replicates were set for each treatment. Ten milliliters of R. solanacearum (1 × 10^6^ CFU mL^−1^) was inoculated to each seedling at the time of transplantation. The control group (CK) was supplied with water. Fulvic acid powder was extracted from yeast molasses fermentation, with 3% N, 0.5% P, 12% K, 6% amino acids, and 60% organic matter (45% fulvic acid). For FA treatment, fulvic acid powder was used with aqueous solution to 150 kg per hectare and 200 mL per plant by irrigating soil around roots. For BP treatment, fermentation broth of *B. paralicheniformis* 285-3 was applied. *B. paralicheniformis* 285-3 was grown in fermentation medium (glucose, 80 g L^−1^; sodium nitrate, 20 g L^−1^; L-sodium glutamate, 20 g L^−1^; sodium citrate, 20 g L^−1^; fulvic acid powder, 20 g L^−1^; and K_2_HPO_4_, 0.5 g L^−1^, pH 6.7) at 37°C and 300 rpm with a ventilatory capacity of 0.48 m^3^ h^−1^ for 48 h (20). The fermentation broth was diluted five times with water, and a 200-mL dilution was applied evenly to the soil around the tobacco roots. For IBP, after 80°C and 30 min of heat-treatment, the fermentation broth was applied in the same manner as above. Two instances of fertilization were applied at 7 days and 25 days posttransplantation for tobacco. The occurrence of tobacco bacterial wilt disease in field was recorded at 60 days posttransplantation, and 30 tobacco plants were selected for each replicate of each treatment (47).

**TABLE 5:** Test treatment design

Treatment	Fertilization conditions
CK	Water
FA	Fulvic acid powder
BP	Bacillus paralicheniformis ferment
IBP	Inactive Bacillus paralicheniformis ferment

### Soil sample collection.

Rhizosphere soil samples were collected respectively at 18 days and 36 days posttransplantation. Eight soil samples were taken separately for each treatment. After removing plant roots and other debris, the soil samples were passed through a 2-mm sieve, then placed in sterile tubes, and quickly put in dry ice and brought back to the laboratory for high-throughput sequencing of soil microorganisms. The soil alkali-hydrolyzable nitrogen (AN), available potassium (AK), available phosphorus (AP), and organic matter (SOM) contents were tested according to previous study (48).

### Rhizosphere microbial community analysis.

DNA was extracted from soil using a FastDNA spin kit (MP Biomedicals, USA) according to the manufacturer's protocol. DNA concentration was determined by a NanoDrop spectrophotometer (Thermo Fisher Scientific, USA) and diluted to 1 ng μL^−1^. Using diluted genomic DNA as the template, specific primers with barcode, Phusion high-fidelity PCR master mix buffer (New England BioLabs), and high-efficiency high-fidelity enzyme were selected for PCR to ensure amplification efficiency and accuracy. The V4 variable region of bacterial 16S rRNA was PCR amplified with primers of 515F (5′-GTGCCAGCMGCCGCGGTAA-3′) and 806R (5′-GGACTACHVGGGTWTCTAAT-3′). The PCR products were purified using magnetic bead. Library construction was performed using the TruSeq DNA PCR-free sample preparation kit. Library was sequenced on the NovaSeq6000 platform.

After the barcode and primer sequences were removed, the reads of each sample were assembled using FLASH. Effective tags were obtained by filtering raw tags. The effective tags were clustered using the UPARSE algorithm and clustering the sequences into OTUs (operational taxonomic units) with 97% identity. Species annotation was performed by Mothur method and SILVA138 database to obtain taxonomic information. Fast multiple sequence alignment was performed using MUSCLE software to obtain phylogenetic relationships of all OTU representative sequences. The data of each sample were normalized, and the sample with the least amount of data was used as the standard. The observed OTUs, Chao1, Shannon, Simpson, and Ace indexes were calculated using QIIME software. The PCoA was performed using R software. The Metastats analysis was performed using the R software. The ANOSIM, MRPP, and Adonis analyses were performed using the R vegan package. AMOVA was performed using the Mothur software.

### Microbial network construction.

The microbial network was constructed using Molecular Ecological Network Analysis Pipeline (MENAP) (28). Using the tobacco disease severity index as the environmental factor, the microbial network was constructed using MENAP and Cytoscape software. First, the OTU abundance was standardized to obtain the standard relative abundance (SRA). The SRA matrix, disease severity index, and OTU annotation information were submitted to MENAP to construct the microbial network. The modules were determined using greedy modularity optimization. The network diagram was formed using Cytoscape 3.9.0 (49). The microbial network was composed of different OTUs (nodes), and the positive or negative interactions between the OTUs were represented by connections (edges) (48). The topology properties and structure of networks were described by modularity, average connectivity (avgK), geodesic distance (avgGD), and clustering coefficient (avgCC). Connectivity represents the number of links between a node with other nodes. Geodesic distance represents the shortest path of two nodes. Clustering coefficient describes how well a node is connected with the neighbor nodes (11).

Topological roles of nodes were defined by within-module connectivity (*Zi*) and connectivity among modules (*Pi*) (13). *Zi* represented how well a node connected to other nodes within its own module. *Pi* represented how well a node connected to other modules. The nodes were divided into four categories according to *Zi* and *Pi* values. Peripheral nodes (specialists) had low *Zi* and *Pi* values that had few links and almost always connected to the nodes within their own modules. Connectors (generalists) had low *Zi* but high *Pi* values that had many links to the nodes within other modules. Module hubs (generalists) had high *Zi* but low *Pi* values that had great links to many nodes in their own modules. Network hubs (supergeneralists) had high *Zi* and *Pi* values that had great links to the nodes both in their own modules and other modules. Peripheral nodes were specialists. Connectors (generalists), module hubs (generalists), and network hubs (supergeneralists) were the keystone microorganisms.

### Statistical analysis.

One-way analysis of variance was performed using SPSS software to test all parameters at the 0.05, 0.01, 0.001, and 0.0001 significance levels. The differences in alpha diversity and beta diversity index between groups were analyzed by Tukey and Wilcox tests using R software. Species with significant differences between groups were analyzed by *t* test using R software.

### Data availability.

The data sets generated and analyzed during the current study are available in the NCBI Sequence Read Archive (SRA) under the BioProject number PRJNA881714.
