# Supplementary material for: Cooperative Action of Fulvic Acid and Bacillus paralicheniformis Ferment in Regulating Soil Microbiota and Improving Soil Fertility and Plant Resistance to Bacterial Wilt Disease
Source: Microbiol Spectr. 2023 Mar 2;11(2):e04079-22. doi: 10.1128/spectrum.04079-22 (PMC10100657; doi:10.1128/spectrum.04079-22)

Table S1 Statistics analysis of Alpha diversity of bacteria at 18 days post-transplantation

Treatment	OTUs number	Shannon	Simpson	Chao1	ACE
IBP18	3652.9±129.4 a	9.6±0.2 a	0.994±0.002 a	4042.1±150.8 a	4094.9±150.4 a
BP18	3670.3±158.9 a	9.5±0.4 a	0.994±0.004 a	4030.6±179.0 a	4105.0±171.0 a
FA18	3645.5±142.7 a	9.5±0.2 a	0.994±0.002 a	4055.9±176.8 a	4113.3±161.4 a
CK18	3414.1±535.6 a	9.4±0.5 a	0.995±0.002 a	3805.5±589.1 a	3865.5±598.0 a

The different letters in the same column represent significant difference between treatments.

IBP18: soil treated with inactive *Bacillus paralicheniformis* ferment at 18 days post-transplantation; BP18: soil treated with *B. paralicheniformis* ferment at 18 days post-transplantation; FA: soil treated with fulvic acid powder at 18 days post-transplantation; CK18: untreated soil as control at 18 days post-transplantation

Figure S1 Venn diagram and dilution curve of soil bacteria in different treatments at 18 days post-transplantation. (A) Venn diagram of bacteria. (B) Rarefaction curve.

A

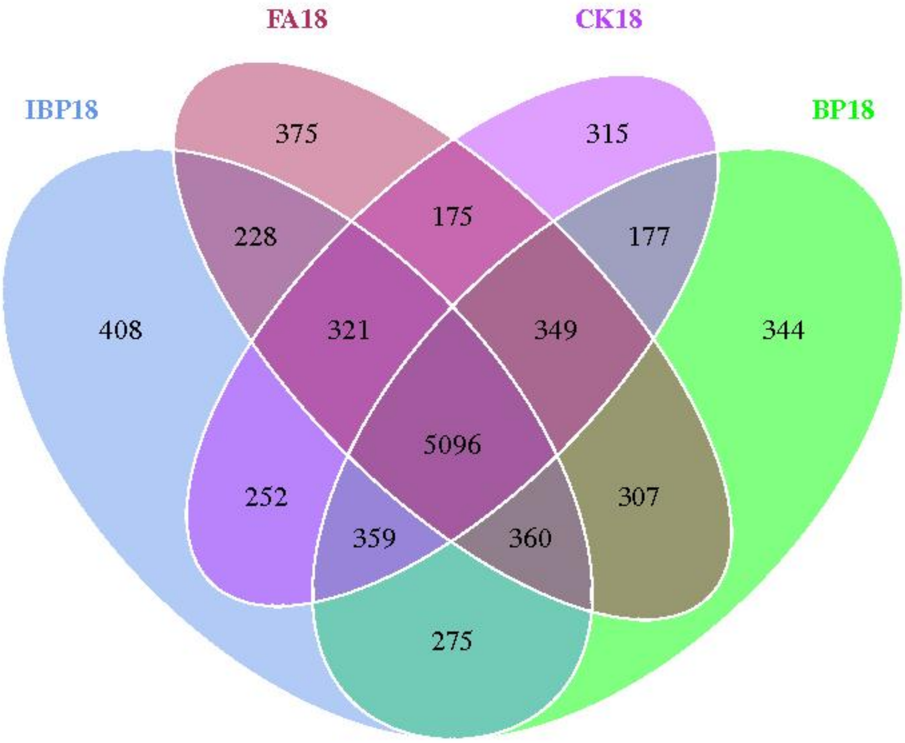

B

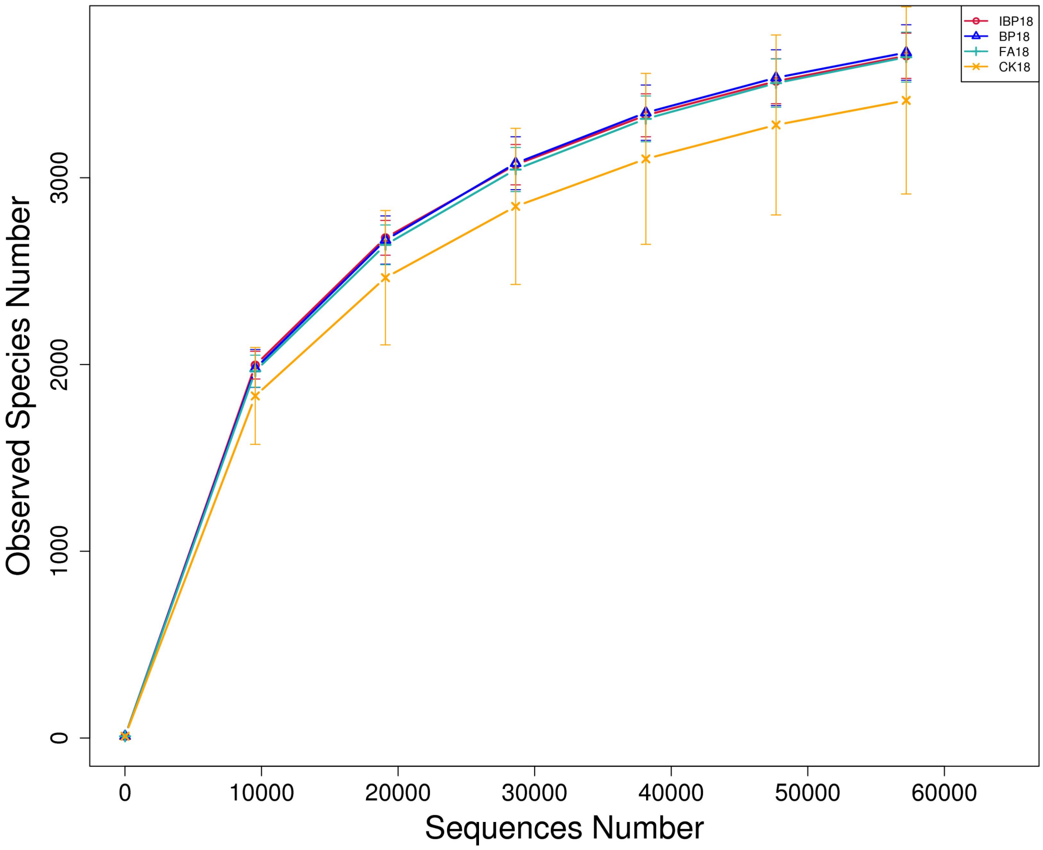

Figure S2 Venn diagram and dilution curve of soil bacteria in different treatments at 36 days post-transplantation. (A) Venn diagram of bacteria. (B) Rarefaction curve.

A

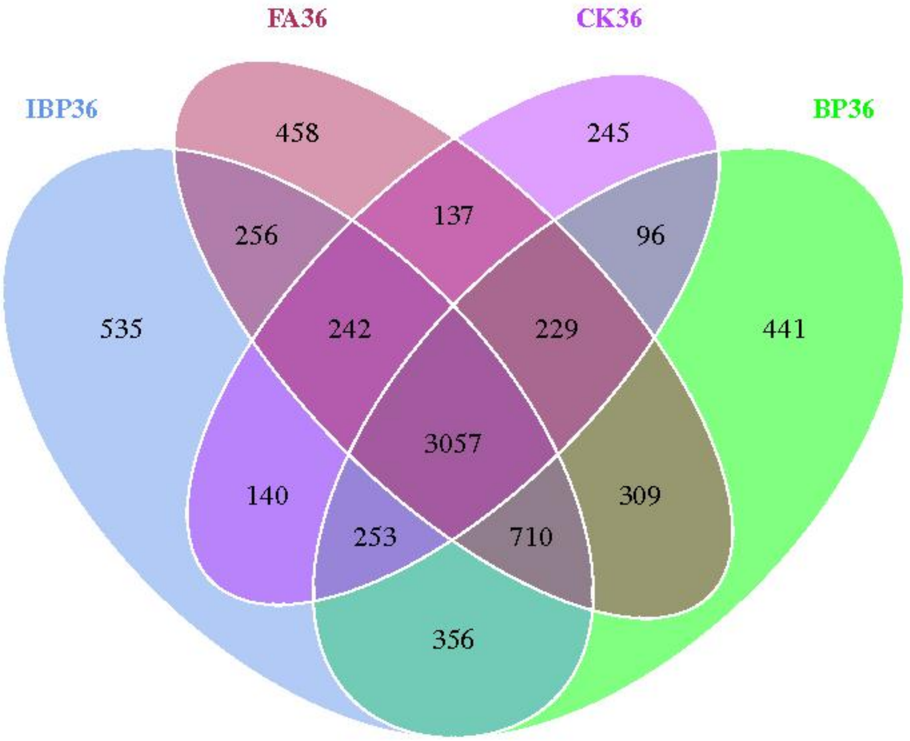

B

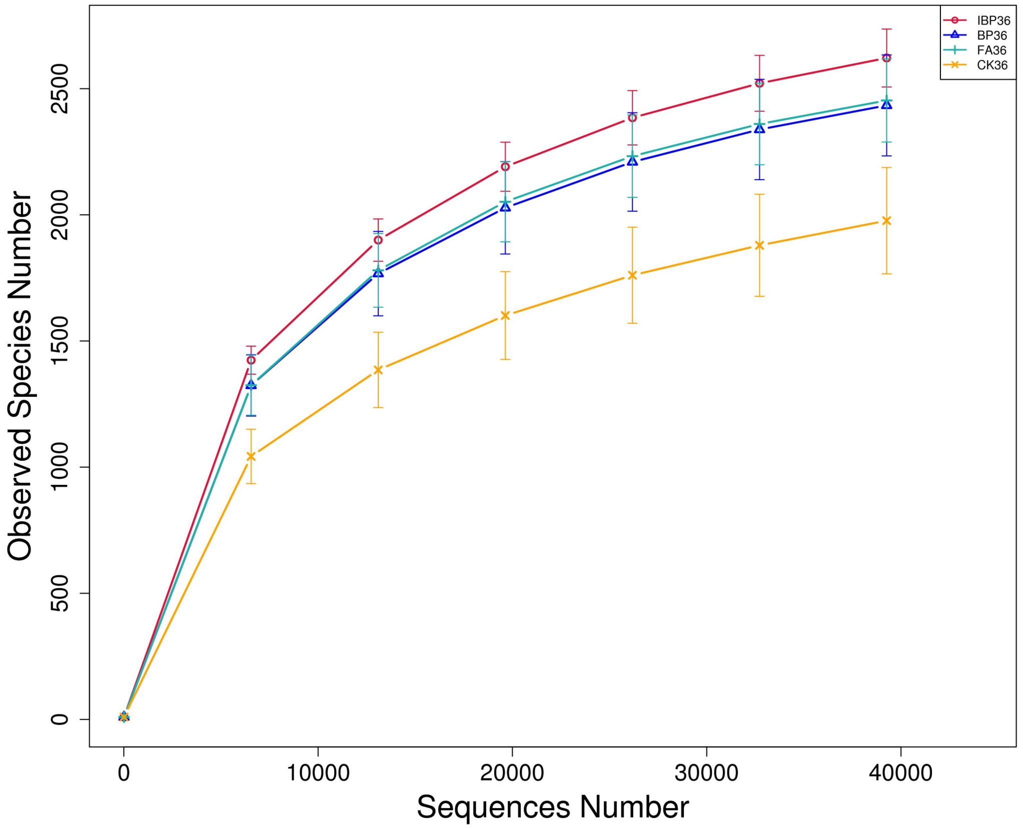

Figure S3 Venn diagram of nodes in microbial networks at 36 days post-transplantation.

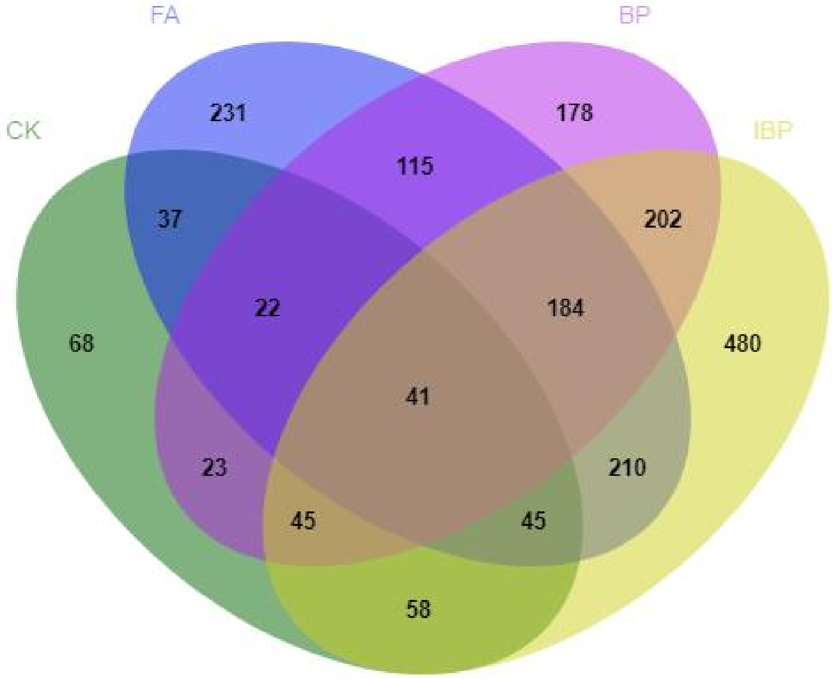

Supplement: Supplemental file 1 — Supplemental material. Download spectrum.04079-22-s0001.pdf, PDF file, 0.7 MB [file spectrum.04079-22-s0001.pdf]
